# The patient and the family: investigating parental mental health problems, family functioning, and parent involvement in child and adolescent mental health services (CAMHS)

**DOI:** 10.1007/s00787-024-02607-3

**Published:** 2024-11-18

**Authors:** Emme-Lina Wirehag Nordh, Karin Grip, Ulf Axberg

**Affiliations:** 1 Department of Psychology, University of Gothenburg, Gothenburg, Sweden; 2 Faculty of Social Studies, VID Specialized University, Oslo, Norway

**Keywords:** Child and adolescent mental health services (CAMHS), Parental mental health problems, Family functioning, Treatment participation

## Abstract

Parental mental health problems can impact child mental health, as well as treatment of child mental health problems. The aim of this study was to investigate self-reported mental health problems in parents of children referred to outpatient child and adolescent mental health services (CAMHS) and to compare child mental health problems, family variables, treatment received and parent participation in treatment planning between parents above and below the cut-off for elevated mental health problems. The sample included *N* = 111 parents of *N* = 98 children. Parental reports covering their own and their children’s mental health problems, family functioning, adult relationship satisfaction, and previous treatment for mental health problems were collected at the time of the child’s intake appointment. Questions concerning contact with CAMHS were completed one year later. At the time of the intake appointment, 44% of the children had a parent who reported elevated mental health problems. In these families, children reportedly had significantly more mental health problems and problematic family functioning was more common than in families where parent mental health problems were not elevated. Parents with elevated mental health problems received group-based parent support/education to a greater extent during the first year of contact with CAHMS and reported less participation in treatment planning compared to parents without elevated mental health problems. To conclude, findings indicate that co-occurring mental health problems are common in parents when their child is referred to CAMHS, and in these families, it is reported that children have more symptoms, and more families experience problematic functioning. These factors merit consideration in assessment and treatment planning, indicating increased treatment needs in this group of families.

Parents’ mental health problems have been found to have consequences for the treatment of their children’s mental disorders. Co-occurring parental mental health problems when their child or adolescent (henceforth “child”) are referred to or have ongoing contact with child and adolescent mental health services (CAMHS), have been associated with higher symptom levels in children at start of treatment [1, 2], increased odds of children being diagnosed with a wide range of psychiatric diagnoses, and an increased risk of psychiatric comorbidity [2], compared with children in CAMHS whose parents are not experiencing mental health problems. Furthermore, initial differences in symptom level have been found to remain after treatment [1], and some studies indicate that children from families experiencing parental mental health problems improve less during treatment [3, 4]. Researchers have therefore recommended considering the parents’ mental health when a child is assessed in CAMHS, to tailor treatment within CAMHS according to child and family needs [5, 6]. Some parents could need or already have their own treatment from other services; and establishing collaboration between adult and child clinical settings could improve treatment of both parental and child mental health problems [5].

Prevalence rates of co-occurring parental mental health problems when their child is in contact with CAMHS vary widely, as rates between 16 and 79% have been reported [5], strongly influenced by study setting and how mental health problems were measured. Estimates based on self-reported mental health problems indicate that about one third of both mothers (36%) and fathers (33%) report elevated mental health problems at the time their child is referred to an outpatient CAMHS unit [7]. Higher prevalence rates have generally been found in studies using reports from professionals about parents’ past or current contact with mental healthcare [5]. A recent Swedish one-day inventory, for example, found that 59% of families with ongoing contact with an outpatient CAMHS unit had at least one more family member in contact with healthcare because of mental health problems, and in 80% of these families the parent was the one needing care [8].

Parental mental health problems, particularly parental mental disorders, are a well-established risk factor for child mental health problems, although the association is complex and influenced by multiple risk and protective factors including both genetic and environmental factors [5, 8]. Child mental health problems can also influence parental mental health. Parenting stress and caregiver strain, referring to a negative psychological response relating to parenthood and additional demands in specific situations, are common in parents whose children need treatment from CAMHS [9, 10]. More parenting stress has in turn been associated with more parental mental health problems [11]. In many families in contact with CAMHS where both child and parent experience mental health problems, many other difficulties in the family context have also been reported, for example, poor or minimal social support, relationship difficulties, and problematic family functioning [5]. Family functioning refers to, for example, how families meet the needs of family members and how conflicts are handled [12]. Problematic family functioning is common in families experiencing parental mental health problems [13] and different aspects of problematic family functioning have also been related to child mental health problems [13]. This makes family functioning important to address in families in contact with CAMHS experiencing co-occurring mental health problems, to better understand their treatment needs.

Within CAMHS, parents are an important part of the treatment of child mental disorders, and their involvement is essential. Parents are usually the ones who identify their children’s difficulties, initiate contact with health services, and facilitate attendance [14, 15]. They are often directly involved in treatment decisions, concerning both treatment options and setting treatment goals, and they are also directly involved in many interventions [14, 15]. Including parents in care decisions has been found to improve their understanding of child difficulties [14] and to be associated with parents’ engagement in interventions [16] and improved treatment outcomes [17]. Barriers to participating in decisions have been explored in many care settings. In paediatric clinical settings, parents’ negative emotional state has reportedly hindered parents’ inclusion and participation in shared decision making [18], as also indicated in a CAMHS population [19]. Further investigation of parents’ participation in decisions in CAMHS, and specifically in parents experiencing mental health problems in this setting, could improve knowledge of how these parents perceive participation.

Although recommended by researchers [5, 20], the identification of parental mental health problems is described as uncommon in clinical practice in child-oriented settings [21], and in a Nordic clinical context, CAMHS medical records usually do not include information about parental mental disorders [2]. Furthermore, Swedish clinical guidelines provide few recommendations for professionals about interventions for families experiencing co-occurring parental and child mental health problems in CAMHS [22]. Taken together, this indicates a need to further investigate parental mental health problems and families in CAMHS experiencing co-occurring parental and child mental health problems, to improve knowledge of needs, associated difficulties, and current clinical practice for these families. In a Swedish context, parental mental health problems in CAMHS have been investigated using reports from professionals [23] and medical records [24], however no previous study has explored parents’ self-reported mental health problems when their child has been referred to CAMHS.

## Aims

The overall aims of the study were to investigate: mental health problems in parents of children referred to outpatient CAMHS, as well as child and family difficulties, and parents’ involvement in treatment. The more specific aims were: (1) to investigate parents’ self-rated mental health problems and previous treatment for their own mental health problems; and to compare parents above and below the cut-off for elevated self-rated mental health problems concerning (2) child symptoms, family functioning and relationship satisfaction, (3) type of contact with CAMHS and (4) perception of participation in treatment planning.

## Method

This longitudinal study has investigated questionnaire data completed by parents who were recruited when their child was referred to CAMHS. The study setting was an outpatient CAMHS unit located in an urban area in Sweden, comprising both a large city and smaller surrounding municipalities. CAMHS offer psychological assessment and treatment for children under 18 years of age with moderate to severe psychiatric problems, including depression, anxiety, ADHD, autism spectrum disorder and behavioral disorders. Treatment options include individual, or group based psychological treatment for the child, individual or group based parental support and/or education as part of the child’s treatment, family meetings and medication. After the initial assessment, treatment options are recommended based on type of difficulties and family needs and a care plan is made in collaboration with the family. The study was run in collaboration between University of Gothenburg and Sahlgrenska University Hospital.

### Participants

All parents of children (*N* = 1824) scheduled for an intake appointment during the recruitment period were invited to participate in this study. The only exclusion criterion applied was parental inability to complete a questionnaire independently in Swedish. Parents of 148 children were interested in participating and the final sample comprised 111 parents (88 women, 23 men) of 98 children (49 girls, 46 boys and 3 other), both of whom participated for 13 children. The included sample correspond to 5% of all eligible children.

The mean age of the participating parents was 45 years (*SD* = 6.3, range 30–68). According to Hollingshead Four Factor Index [25], most participating parents were middle or high in social status (*n* = 96, 87%); and in comparison with a population-based reference group (*n* = 226, *M* = 38.2, *SD* = 11.7) [26], the social status in the included sample (*n* = 111, *M* = 46.5, *SD* = 13.5) was statistically significantly higher (*t*(335) = 5.83, *p* < .001, *d* = 0.68, 95% CI [0.44, 0.91]). The mean age of the children in the sample was 12.5 years (*SD* = 3.1, range 5–17). The reasons for contact with CAMHS were mainly related to neurodevelopmental, depressive, or anxiety disorders (for details on participant characteristics, see Table 1).

**Table 1:** Sociodemographic characteristics of parents (*N* = 111) and children (*N* = 98)

Characteristic	
Parents	
***Age***,*** years***, **M (SD) [range]**	45 (6.3) [30–68]
***Gender***, **n (%)**	
Female	88 (79)
Male	23 (21)
***Country of birth***, **n (%)**	
Sweden	91 (82)
Nordic countries (except Sweden)	3 (3)
Europe (except Nordic countries)	5 (5)
Outside Europe	12 (11)
***Civil Status***, **n (%)**	
Married or cohabiting	85 (77)
In a relationship but living apart	8 (7)
Single	17 (15)
Other	1 (1)
***Hollingshead Social Status***, **M (SD) [range]**	46.5 (13.5) [11–66]
Low, < 30, n (%)	15 (14)
Middle–high, ≥ 30, n (%)	96 (86)
**Children**	
***Age***,*** years***, **M (SD) [range]**	12.5 (3.1) [5–17]
***Gender***, **n (%)**	
Female	49 (50)
Male	46 (47)
Other	3 (3)
***Country of birth***, **n (%)**	
Sweden	96 (98)
Outside Europe	2 (2)
***Custody***, **n (%)**	
Joint	84 (86)
Sole	13 (13)
Other	1 (1)
***Living arrangements***, **n (%)**	
With primary informant and partner/husband/wife	64 (65)
Alternating between separated parents ^a^	12 (12)
Mainly with primary informant	12 (12)
Only with primary informant	6 (6)
Out-of-home care	1 (1)
Other	3 (3)
***Reason of contact with CAMHS***, **n (%)**	
Neurodevelopmental disorders (ADHD, autism spectrum and related disorders)	46 (47)
Depressive disorders	19 (19)
Anxiety disorders, obsessive–compulsive and related disorders	12 (12)
Eating disorder	3 (3)
Disruptive, impulse-control, and conduct disorders	4 (4)
Deliberate self-harm	4 (4)
Other (family relations, school difficulties)	6 (6)
Missing	4 (4)
***Previous contact with professionals due to mental health problems***, **n (%)**	
Yes	91 (93)
No	7 (7)
***If yes***,*** which services***, **n (%) (multiple choices possible)**	
School healthcare	54 (55)
Primary healthcare centre	42 (43)
Medical centre for children and adolescents	21 (21)
Habilitation centre for children and adolescents	10 (10)
Youth guidance centre	7 (7)
Private options (assessment or treatment)	13 (13)
Social services	22 (22)
Another CAMHS unit	5 (5)
Other	8 (8)

*Note*. The percentages do not sum to 100 for *parents’ country of birth* and *living arrangements* due to rounding

^a^ Living equal amounts of time with both of their separated parents

### Procedure

Participants were recruited from January 2020 through February 2021. All legal guardians received brief written information about the study along with information about the intake appointment at CAMHS. Parents interested in participating contacted the research team via an accessible QR link and were sent more information about the study. Parents choosing to participate completed a web-based questionnaire, estimated to take 30–45 min, at the time of the intake appointment. Upon request, the questionnaire could be completed on paper. One year later, parents were contacted via email with information and a link to a follow-up questionnaire, including questions concerning their contact with CAMHS during the year after their first appointment.

The study mainly took place during the peak of the COVID-19 pandemic. During spring 2020 many appointments were rescheduled due to restrictions and the contact could in some cases take more time before it started, however care was provided based on psychiatric needs and type of interventions offered did not change. During this period there was however a transition into using more digital options to complement face-to-face visits.

### Measures

The questionnaires comprised measures covering parental and child mental health problems, family functioning, adult relationship satisfaction, as well as questions concerning background and contact with CAMHS, as follows.

The ***Symptom Checklist (SCL-90)*** [25] is a widely used measure of self-reported psychiatric and somatic symptoms comprising 90 items rated on a five-point scale (0–4), with higher scores indicating more symptoms. The Swedish version of SCL-90 has been found valid and reliable [26]. In this study, the Global Severity Index (GSI, range 0–360) including all items was used to assess the level of mental health problems in parents. To adjust for age and gender, the GSI raw scores were transformed into T scores [26]. A T score ≥ 63, corresponding to the 90th percentile, was used as a cut-off to reflect elevated scores. The Cronbach’s α was 0.98.

Parents were also asked whether they had previously received treatment for mental health problems during their child’s life and, if so, in what service setting(s), for what problem(s), and what type(s) of treatment they had received. The parents were also asked whether they thought the child’s other parent had ever experienced mental health problems during the child’s life. The questions concerning the parents’ own previous treatment and perception of the other parent’s mental health were combined to estimate how many children had experienced mental health problems in neither, one, or both parents during their life.

The ***Total Difficulties Score*** of the ***Strength and Difficulties Questionnaire—Parent version (SDQ-P)*** [27, 28] was used to measure parents’ ratings of child mental health problems. It includes 20 items responded to on a three-point scale (0–2) concerning emotional symptoms, conduct problems, hyperactivity–inattention, and peer problems. The Total Difficulties Score ranges from 0 to 40, with higher scores indicating more problems. The Swedish version has shown satisfactory psychometric properties [28]. The Cronbach’s α was 0.79.

The ***General Functioning Subscale*** of the ***Family Assessment Device (FAD-GF)*** [13, 29] was used to assess the parents’ perception of family functioning. The subscale comprises 12 items responded to on a four-point scale (1–4). Higher scores represent more problematic functioning concerning problem solving, communication, roles, affective responsiveness and involvement, and behavioural control. The subscale has been found to have adequate psychometric properties [13]. A cut-off score ≥ 2.1 was used to indicate problematic functioning [29]. The internal consistency of this scale was indicated by a Cronbach’s α of 0.90.

***Dyadic Adjustment Scale***, ***brief version (DAS-4)*** [30] was used to measure adult relationship satisfaction in currently partnered parents. The measure includes four questions concerning how often divorce or separation is discussed, whether the relationship is going well, how much the partners confide in each other, and happiness level. Questions are rated on scales of 0–5 or 0–6, with higher scores indicating more satisfaction. The summary scale ranges from 0 to 21, and a score < 13 was used to indicate relationship distress [30]. The internal consistency of this scale was indicated by a Cronbach’s α of 0.84.

***Hollingshead Four Factor Index of Social Position*** [31] was calculated based on questions concerning occupation and profession, with higher scores indicating higher social status (range 8–66). Scores were dichotomized into low (< 30) and medium–high (≥ 30) and a Swedish population-based reference group was used for comparison [32].

***Questions about the contact with CAMHS*** concerned if the child was still a patient one year later and if so, the current intensity of the contact. Furthermore, parents were asked about the type of interventions the children and/or family had received during the first year of contact (i.e., psychological assessment and/or treatment); for those who had received treatment, parents were asked to respond with a yes or no if they had received different treatment options (i.e., individual or group-based child treatment, individual or group-based parent support (training/education), family meetings or child medication). Parents were also asked about whether their own mental health problems had been addressed in conversations with the mental health professionals during their contact with CAMHS, and if they would have liked to talk less, were satisfied, or would have liked to talk more about it.

Four questions from the ***Ohio Youth Scales*** [33] were used to measure parents’ perception of overall satisfaction with mental health services (one question) and participation in treatment planning (three questions). The responses range from 1 to 6, with higher scores indicating less satisfaction/participation. The four items are summed to a total score, for which the Cronbach’s α was 0.93. We added one additional question concerning the parents’ perception of their child’s participation in treatment planning which was not included in the total score.

***Background questions*** about the parent concerned age, gender, country of origin, civil status, and education and about the child concerned age, gender, legal custody, living arrangement, reason for contact with CAMHS, and previous professional contacts because of mental health problems.

### Data analysis

Data were examined for outliers and normality. To reduce the influence of one extreme outlier on SCL-90 (*SD* > 3.29), the Winsorizing procedure was used [34]. Data were found to be normally distributed for SDQ-P and moderately positively skewed for DAS-4, FAD-GF, and SCL-90 (T scores), so the assumptions for using parametric tests were considered fulfilled. According to Little’s MCAR test, data was missing completely at random. Missing data were handled according to guidelines for the included measures, specifying how many items could be missing when calculating subscale and total scale scores. Recommendations were unavailable for DAS-4, so we decided that all items needed to have been completed to compute a total scale score. When all scale scores were summed, under 2% of the values were missing.

In families where both legal guardians participated in the study, information from one primary informant (mothers *n* = 84, fathers *n* = 14) was used for reports about the child and family. When available, the mothers were chosen as primary informants, as they were more likely to participate in the study.

Independent-samples *t*-tests were used for mean comparisons between groups, and the Pearson chi-square test and Fisher exact test was used to determine whether proportions were equal between groups. For statistical analysis, we used SPSS version 28.0 and Laken’s Excel sheet for statistical analysis [35]. Two-tailed *p*-values < 0.05 were considered statistically significant. Cohen’s *d* was used to report effect sizes.

### Ethics

The study was reviewed and approved by the Regional Ethical Review Board in Gothenburg (reg. no. 806 − 17). Ethical considerations about asking parents to participate in a study of their own mental health problems in a service setting where they were not themselves identified as patients were taken into account. The risks of asking questions concerning the parent’s own mental health problems and family functioning were considered low, as parents were in contact with professionals who could support them or guide them where to seek help if needed. Furthermore, questions concerning heredity and the family situation are usually asked early in contacts with CAMHS. Parents consented to participate in the questionnaire.

### Attrition

Child age and gender in the study sample did not differ significantly from those of all children whose parents had been invited to participate. At follow-up, parents (*n* = 80) of 71 children completed the questionnaire. Social status according to the Hollingshead Four Factor Index was found to be significantly higher among those parents who completed the follow-up questionnaire (*t*(109) = 3.10, *p* = .002, *d* = 0.66, 95% CI [0.23, 1.08]). No other differences were found.

## Results

### Parental mental health problems

At the time of the child’s first assessment at CAMHS, 44% (*n* = 43) of the included children had parents who reported elevated levels of mental health problems on SCL-90 GSI (T-score ≥ 63). Furthermore, 48% (*n* = 47) of the children had parents who reported having received treatment for own mental health problems during the child’s life. Most commonly, parents had received treatment from primary care, private options (e.g., psychologist, psychotherapist, or occupational healthcare), or secondary outpatient care (see Table 2). Stress-related disorders (primarily exhaustion disorder), depression, and anxiety disorders were the most frequently stated reasons for treatment contacts.

**Table 2:** Parent’s (*N* = 111) own previous treatment for mental health problem during the child’s life and perception of the other parent’s mental health problems during the child’s life

Variable	*n* (%)
**Previously received treatment for own mental health problems?**	
No	64 (58)
Yes	47 (42)
**For those who answered yes**:	
***When did you receive treatment?***	
During the past three months	15 (32)
During the past year	8 (17)
During the past 18 months	4 (9)
Further back in time than 18 months	20 (43)
***For what type of mental health problem(s)?*** **(Multiple choices possible)**	
Neurodevelopmental disorders (ADHD, autism spectrum and related disorders)	4 (9)
Bipolar disorder	1 (2)
Depressive disorders	26 (55)
Anxiety disorders, obsessive–compulsive and related disorders	12 (26)
Trauma and stressor-related disorders	
PTSD	6 (13)
Other reactions to severe stress (work-related)	27 (57)
Substance-related and addictive disorders	1 (2)
Personality disorders	1 (2)
Other	7 (15)
***From which service setting(s)?*** **(Multiple choices possible)**	
Primary care	33 (70)
Private options	15 (32)
Secondary care (outpatient)	10 (21)
Secondary care (inpatient)	3 (6)
Social services	1 (2)
***Type(s) of treatment(s)?*** **(Multiple choices possible)**	
Medication	25 (53)
Individual therapy	36 (77)
Group therapy	1 (2)
Family therapy	1 (2)
Physiotherapy	5 (11)
Occupational therapy	4 (9)
Other (e.g., sick leave, rest)	4 (9)
***All parents were also asked about if the other legal guardian had experienced mental health problems during the child’s life***	
No	57 (51)
During the past three months	8 (7)
During the past year	8 (7)
During the past 18 months	6 (5)
Further back in time than 18 months	25 (23)
Not applicable, only one parent	7 (6)

Self-reported mental health problems at the time of the first appointment were significantly higher in parents who reported having previously received their own treatment (*M* = 67.7, *SD* = 15.0), versus those who had not (*M* = 57.5, *SD* = 15.0; *t*(109) = 3.56, *p* < .001, *d* = 0.68 [0.30, 1.07]). Of the parents reporting elevated mental health problems, 42% (*n* = 19) reported not having received previous treatment for mental health problems.

Based on the question about the parent’s own previous treatment contact and the question about their perception of the other parent’s mental health, it was estimated that 50% (*n* = 49) of the included children had experienced parental mental health problems during their life, 16% (*n* = 16) in both parents, and 34% (*n* = 33) in one parent.

### Comparison between parents above and below the cut-off for elevated mental health problems

#### Child symptoms

Children of parents with elevated self-rated mental health problems reportedly had significantly higher levels of symptoms on SDQ-P (*M* = 20.6, *SD* = 5.5) than did children of parents below the cut-off (*M* = 15.3, *SD* = 6.5; *t*(96) = 4.32, *p* < .001, *d* = 0.88, 95% CI [0.46, 1.30]).

#### Family functioning

General family functioning was rated as problematic in 46% (*n* = 45) of the included families, and the proportion of families with problematic family functioning was significantly higher among families where parents reported elevated mental health problems, than in those below the cut-off (χ^2^ (1, *N* = 97) = 9.54, *p* = .002).

#### Relationship satisfaction

Relationship satisfaction was reported by parents currently in a relationship, and dissatisfaction (score < 13) was reported in 27% of these families. The proportion of parents reporting relationship dissatisfaction did not differ between families where parents scored above or below the cut-off for elevated mental health problems.

#### Contact with CAMHS

At follow-up, 49% (*n* = 37) of the parents whose children had received treatment reported that their own mental health had been addressed in conversations with the mental health professionals. About half of these parents (*n* = 20, 54%) wished that their own mental health problems had been discussed more, 43% (*n* = 16) were satisfied with the extent of discussion, and one (3%) thought that his/her mental health problems had been discussed too much. Of parents who reported elevated mental health problems at the time of the first appointment, 50% (*n* = 13) stated that their mental health had not been addressed and 35% (*n* = 9) had discussed their mental health but wished it had been discussed more.

When different types of treatment were compared, we found that parents over the cut-off reported having received group-based parent training/education to a greater extent during the first year of contact with CAMHS (χ^2^ (1, *N* = 65) = 5.96, *p* = .019). We found no other statistically significant differences concerning contact with CAMHS or treatment options received (see Table 3).

**Table 3:** Parent’s descriptions of interventions received from CAMHS during the first year of contact and perceived satisfaction with and participation in treatment planning

Variable	Full sample	Parents under cut-off ^a^	Parents over cut-off ^a^	*p*
***Contact one year after the first appointment***,*** n*(%)**				
No longer in contact with CAMHS	17 (24)	13 (28)	4 (16)	
2–4 meetings per month	17 (24)	12 (26)	5 (20)	0.533
1 meeting per month or less	26 (37)	15 (33)	11 (44)	
Other	11 (16)	6 (13)	5 (20)	
***Type of interventions during the first year***,*** n*(%)**				
No intervention received	3 (4)	2 (4)	1 (4)	
Psychological assessment only	2 (3)	2 (4)	0 (0)	
Psychological assessment and treatment	38 (50)	21 (47)	17 (68)	0.327
Treatment only	27 (36)	20 (44)	7 (28)	
***Were parent mental health problems addressed by professionals? n*** **(%)**				
No	38 (51)	25 (51)	13 (50)	
Yes	37 (49)	24 (49)	13 (50)	
***If yes***,*** were the parent satisfied with the extent to which it was addressed? n*(%)**				
Would have wanted to talk more about it	20 (54)	11 (46)	9 (69)	
Satisfied with the extent	16 (43)	12 (50)	4 (31)	
Would have wanted to talk less about it	1 (3)	1 (4)	0 (0)	
***Type of treatment received from CAMHS*(multiple choices possible)**, ***n*(%)**^**b**^				
Child individual treatment	39 (60)	26 (63)	13 (54)	0.601
Child medication	38 (59)	22 (54)	16 (67)	0.435
Parent individual support	11 (17)	6 (15)	5 (21)	0.733
Parent group-based support	16 (25)	6 (15)	10 (42)	0.019*
Family meetings	12 (19)	5 (12)	7 (29)	0.108
Other (e.g., occupational therapy, dietician support, and support in school)	14 (22)	11 (27)	3 (13)	0.222
***Ohio satisfaction scale***, **M (SD)**				
Total score (sum of question 1–4)	13.0 (7.8)	12.1 (4.6)	14.8 (5.5)	0.031*
1. Satisfaction with services received	3.4 (1.4)	3.3 (1.3)	3.7 (1.4)	0.188
2. Parent included in treatment planning	3.0 (1.5)	2.8 (1.4)	3.6 (1.6)	0.047*
3. Professionals listened to and valued the parent’s ideas	3.2 (1.3)	3.0 (1.2)	3.7 (1.5)	0.022*
4. To what extent does the treatment plan include the parent’s ideas	3.4 (1.2)	3.2 (1.2)	3.8 (1.3)	0.035*
5. Child included in treatment planning	3.6 (1.4)	3.4 (1.5)	4.0 (1.4)	0.139

*Note*. Pearson Chi-square for comparison of contact one year later and type of treatment received, Fisher Exact test for contact consisted of and independent-samples *t*-test for Ohio satisfaction scale

^a^ Parents under or over T score of 63 on SCL-90 GSI = Symptom Checklist 90 Global Severity Index

^b^ Numbers indicate those who answered yes, of those who received treatment from CAMHS. No children had received group treatment

* *p* < .05 (two-tailed)

#### Perceived satisfaction and participation in treatment planning

Satisfaction with the mental health services and perceived participation in treatment planning varied greatly between parents (see Table 4). Parents with mental health problems over the cut-off at the time of the child’s intake appointment were found to report statistically significantly less perceived participation in treatment planning one year later than did those below the cut-off (see Table 3).

**Table 4:** Parent’s perceived satisfaction with mental health services and perceived participation in treatment planning reported at follow-up one year after the first appointment at CAMHS

Question	*N*	Response alternatives, *n* (%)					
		Completely satisfied	Moderately satisfied	Somewhat satisfied	Somewhat dissatisfied	Moderately dissatisfied	Extremely dissatisfied
1. How satisfied are you with the mental health services your child has received so far?	64	0 (0)	20 (31)	20 (31)	7 (11)	10 (16)	7 (11)
		**A great deal**	**Moderately**	**Quite a bit**	**Somewhat**	**A little**	**Not at all**
2. To what degree have you been included in the treatment planning process for your child?	65	12 (19)	14 (22)	13 (20)	16 (25)	5 (8)	5 (8)
3. Mental health workers involved in my case listen to and value my ideas about treatment planning for my child	64	4 (6)	19 (30)	14 (22)	17 (27)	5 (8)	5 (8)
4. To what extent does your child’s treatment plan include your ideas about your child’s treatment needs?	64	2 (3)	13 (20)	18 (28)	19 (30)	7 (11)	5 (8)
5. To what degree has your child been included in the treatment planning process?	64	4 (6)	10 (16)	14 (22)	22 (34)	7 (11)	7 (11)

*Note*. Questions 1–4 are from the OHIO satisfaction scale, and question number 5 was added by the authors

## Discussion

This study has investigated mental health problems in parents of children referred to an outpatient CAMHS unit. Furthermore, parents above or below the cut-off indicating elevated mental health problems at the time of the child’s first appointment were compared concerning child mental health problems, family variables, and parents’ involvement in treatment. In our sample, 44% of the children had parents who reported elevated mental health problems, and in these families, children reportedly had statistically significantly higher levels of symptoms and a higher proportion of families had problematic family functioning. We also found that parents with elevated mental health problems to a greater extent reported having received group-based parent support/education during the first year of contact with CAMHS and reported significantly less perceived participation in treatment planning.

The results indicate that in many families referred to CAMHS, both child and parent(s) experience mental health problems, implying that co-occurring mental health problems are often dealt with by mental health professionals in CAMHS in their everyday practice. In our study, parental mental health problems were assessed in two ways, boththrough self-reports using a dimensional measure of symptoms and by asking parents whether they had previously received treatment for mental health problems during their child’s life. Together, the results indicate that past and current mental health problems are common in this group of parents. The results concerning self-rated parental mental health problems, showing that 44% of the children in our sample had at least one parent who reported elevated mental health problems, are in line with previous research in similar clinical settings, where it has been reported that 36% of mothers and 33% of fathers report elevated psychiatric symptoms at the time of their child’s intake appointment [7]. The finding that 48% of children in our sample had at least one parent who reported to have received services for mental health problems during the child’s life, are furthermore in line with Swedish studies using professionals’ reports and medical records, indicating that 59% of families have more than one family member in need of mental health care [23] and that about half of the children between 12 and 18 years in contact with CAMHS had a parent with a previous contact with primary or secondary mental health care [24]. The prevalence rates found in our study must, however, be interpreted cautiously, as the included families represent only a small sample (5%) of those eligible. Furthermore, we found that the participating parents had significantly higher social status compared with a population-based reference group. Based on the questions on the measure we used, this indicate that parents in our study had higher education level and/or higher status positions than parents in the general population [25]. Furthermore, sick-leave or unemployment markedly lower social status on this measure. Taken together, this indicate that families with socioeconomic difficulties may not be represented in our sample. From other studies it has been reported that families experiencing parent mental illness are families with a higher risk of socioeconomic adversity [36], which further point in the direction that we in our sample have a group of parents and families where parents with higher levels of mental health problems may not be represented. This indicate that the prevalence rates found in our study may be an underestimation of mental health problems in parents of children referred to CAMHS. We also lost parents with elevated mental health problems to attrition, and thus have information from parents with lower levels of mental health problems at follow up.

During initial assessment in CAMHS, questions concerning the heredity of psychiatric disorders and the family situation are usually asked [22]. The results from the present study suggest the importance of also including questions about co-occurring parental mental health problems during this assessment. If only questions concerning heredity are asked, families with co-occurring mental health problems may not be identified. In fact, in our sample we found that among those parents who reported elevated mental health problems, many (42%) had no previous treatment contact with mental health services. Furthermore, at follow-up, half of parents with elevated mental health problems at intake reported not having discussed their own mental health problems during the contact with CAMHS. Asking about parental mental health status is recommended by researchers, who argue for the importance of including this information to guide treatment planning [5, 20, 37] and in our results many parents were satisfied with how much their mental health had been discussed or would have liked to discuss it more. Assessing parental mental health problems in CAMHS, however, has ethical implications, as parents are not the patients in this service setting, and what should be included in such an assessment needs careful consideration [21]. Nevertheless, as a way of gathering information to guide clinical decisions in CAMHS, this matter deserves consideration.

The present findings also revealed that children of parents with elevated mental health problems reportedly had significantly higher levels of mental health problems. Associations between higher parental mental health problems and more child symptoms have previously been demonstrated [2, 5], underscoring that these families have complex care needs. Other studies have, for example, found that children from families with co-occurring parental and child mental health problems receive longer treatment in CAMHS [2]. Furthermore, our findings showed that problematic family functioning was more common in families experiencing parental mental health problems, suggesting that aspects of family functioning could be important to address during assessment and treatment in CAMHS to support these families. Difficulties in the family context has also been reported in several other studies into families experiencing co-occurring parent and child mental health problems [5].

Supporting parents within CAMHS is important, as they are central to their child’s environment and may be an important part of their child’s treatment. Furthermore, parents are sometimes the main treatment target, for example, in parent training programmes [14]. We found that parents with elevated symptoms reported having received group-based parent training/education to a greater extent. This could be important to consider when giving these interventions in CAMHS, as previous research has shown that maternal depressive symptoms, for example, can negatively influence engagement in treatment and completion of parental support programmes [38]; notably, participating in parental support groups has also been observed to have positive effects on parental mental health problems, for example, depressive symptoms [39]. Considering the parent’s mental health is nevertheless important to take into account when deciding on treatment options, as families with co-occurring parent mental health problems could be in need of more tailored interventions from CAMHS based on their more complex needs.

Some parents might also need their own treatment from other services [40], and researchers have suggested that collaboration between child- and adult-oriented services concerning treatment coordination should be developed [5]. Others have advocated using treatments targeting both child and parental mental health problems simultaneously, although more research is needed concerning such interventions [41]. This points to challenges concerning how to organize care when multiple family members experience mental health problems, and finding ways to reduce barriers to seek and receive care for the whole family is essential.

Parents are also directly involved in decisions in CAMHS. The positive impact of participating in decisions and being able to influence treatment [14] emphasizes the need to improve participation in these families, as parents reporting elevated mental health problems reported less perceived participation in treatment planning. From previous research in paediatric settings and CAMHS it has been reported that parent’s or child’s negative emotional state can be a barrier for shared decision making as they might be overwhelmed, anxious or not accepting the situation, resulting in that they were not included or do not want to be a part of the decision process [18, 19]. This could be factors that explain difference found also in our sample. Involving parents in treatment planning decisions could be one way of supporting parents and increasing their engagement, which is essential for effective treatment [42], and point to the importance of finding ways to facilitate shared decision making in families experiencing co-occurring parent and child mental health problems in CAMHS.

## Strengths and limitations

Strengths of this study are its naturalistic design and the informing and inviting of all those eligible to participate. The use of parental reports made it possible to investigate both previous treatment for and current self-reported mental health problems in parents. However, an important limitation is that the included sample was small relative to all parents approached, affecting the generalizability of results. One reason for the low participation could be because the data collection started in January 2020, simultaneously with the onset of the Covid pandemic, and the number of parents interested in participating was particularly low in spring 2020. The recruitment period was accordingly prolonged to 13 months from the initially planned six months. Nevertheless, the results from this study are in line with results from other Swedish studies concerning number of parents with mental health problems in CAMHS [7, 24], which strengthen the conclusions from this study. Another limitation is that the results concerning reason of contact with CAMHS and type of contact during the first year are based only on parental reports, and there can be discrepancies between parent descriptions compared to reports from professionals. Furthermore, a possible risk of bias in parental reports about child mental health problems have been indicated by some studies, i.e., that these parents experiencing mental health problems report more child symptoms than do those who do not, however, the evidence for such a bias is not conclusive and other studies have not found this difference [43].

## Future research

A larger representative sample is needed in order to report the prevalence rates of parental mental health problems in CAMHS. Reviewing medical records and using reports from mental health professionals could improve knowledge of clinical practice concerning the type of treatment received by families experiencing co-occurring mental health problems in this clinical setting. Research into how parental mental health problems as well as family functioning are related to change in child mental health problems over time could improve knowledge of how these variables influence treatment outcomes. Furthermore, interventions and recommendations concerning how to meet the needs of this subgroup of families within CAMHS or in inter-agency collaborations need to be developed.

## Conclusion

Results of this study indicate that many families in CAMHS experience co-occurring mental health problems in children and parents. In these families, children were found to have a higher symptom load and more families reportedly have problematic family functioning. These families constitute a vulnerable subgroup of families in CAMHS with complex and increased care needs that should be identified and appropriately addressed.

## Data Availability

The data that support the findings of this study are not openly available due to reasons of sensitivity and are available from the corresponding author upon reasonable request. Data are located in controlled access data storage at the University of Gothenburg.
